# An Unusual Cause of Adrenal Mass in Neurofibromatosis Type 1: Malignant Peripheral Nerve Sheath Tumor

**DOI:** 10.7759/cureus.21782

**Published:** 2022-01-31

**Authors:** Awni Shahait, Tanya Odisho, Bayan Alshare, Lana J Alghanem, Donald Weaver

**Affiliations:** 1 Surgery, The Michael and Marian Ilitch Department of Surgery, Wayne State University School of Medicine, Detroit, USA; 2 Department of Surgery, Sinai Grace Hospital, Detroit Medical Center, Detroit, USA; 3 Oncology, Barbara Ann Karmanos Cancer Institute, Detroit, USA; 4 Pharmacology, Eugene Applebaum College of Pharmacy and Health Sciences, Wayne State University, Detroit, USA

**Keywords:** adrenal pheochromocytoma, neurofibromatosis 1, malignant, adrenal mass, peripheral nerve sheath tumor

## Abstract

A malignant peripheral nerve sheath tumor (MPNST) is an aggressive tumor that can arise from the malignant transformation of benign neurofibromas in patients with neurofibromatosis type 1 (NF1). MPNST occurs in 2% of patients with NF1, contributing to significant mortality in these patients. Here, we report the case of a 67-year-old female with a known history of neurofibromatosis type 1 who was referred to general surgery after the discovery of a large left-sided adrenal mass on CT imaging five months earlier. Lab workup revealed elevated urine catecholamines, concerning pheochromocytoma. As pheochromocytoma is also common in those with NF-1, appropriate medical management followed by surgical resection was performed. The final pathology report revealed an MPNST.

## Introduction

Neurofibromatosis type 1 (NF1), or von Recklinghausen’s disease, is an autosomal dominant neurocutaneous disorder affecting approximately 1:3000 people worldwide [1]. Patients with NF1 can display a wide range of pathologic abnormalities, but the most common include those of the epidermis (café au lait macules), adrenal gland (pheochromocytoma), brain (astrocytoma), and peripheral nerve sheath (neurofibroma and malignant peripheral nerve sheath tumors [MPNSTs]) [2]. MPNSTs are highly aggressive tumors thought to arise from malignant transformation of benign neurofibromas in patients with NF1 [2]. They occur in approximately 2% of those with NF1 and contribute to a significant number of NF1 related deaths, with a five-year survival rate of 34-52% [2,3].

The most common sites of MPNSTs include the extremities (45-59%), the trunk (17-34%), and the head and neck (18-24%) [3]. The presentation may consist of a rapidly enlarging mass causing pain. Some patients may experience local neurological symptoms such as paresthesia or weakness [4]. Up to 50% of patients have metastatic disease at the time of diagnosis, most commonly to the lungs [4]. 

Differentiation of MPNSTs from other soft tissue tumors on imaging can be difficult. CT usually shows a non-specific soft tissue mass [3]. MRI is considered the most useful imaging modality as it can characterize the extent of tumor involvement in relation to the surrounding anatomy [4]. MPNSTs are fluorodeoxyglucose (FDG)-avid on PET/CT with a mean maximum standardized uptake value (SUV) of 8.5 [3].

The mainstay of treatment for MPNSTs is surgical resection with negative surgical margins before distant metastasis occurs [5]. While chemotherapy and adjuvant radiotherapy are used in many centers as adjuncts to surgical resection, most MPNSTs are considered chemotherapy- and radiotherapy-resistant [5].

This case describes a patient with known NF1 who presented with a symptomatic left adrenal mass. As work up was suspicious for pheochromocytoma, she underwent an open left adrenalectomy. The final pathology revealed MPNST. Diagnostic challenges are common in NF1 patients presenting with an unidentified mass. This case highlights the importance of considering more rare diagnoses, such as MPNST, in individuals with NF1 who are present with adrenal masses.

## Case presentation

A 67-year-old female, with known neurofibromatosis type 1, was referred to general surgery with a left-sided adrenal mass associated with abdominal pain, nausea, and emesis. Medical history includes chronic obstructive pulmonary disease, hypertension, and a remote history of hormone-sensitive breast cancer stage IIb, treated with left modified radical mastectomy followed by chemotherapy. She had presented to the emergency department five months earlier and, following workup, was found to have an incidental left-sided adrenal mass on imaging.

On examination, her blood pressure was 128/62 mmHg with a heart rate of 80 bpm. Innumerable neurofibromas were noted on her face, trunk, and upper and lower extremities. Abdominal examination demonstrated left upper quadrant pain on deep palpation with no palpable masses or organomegaly.

**Figure 1:**
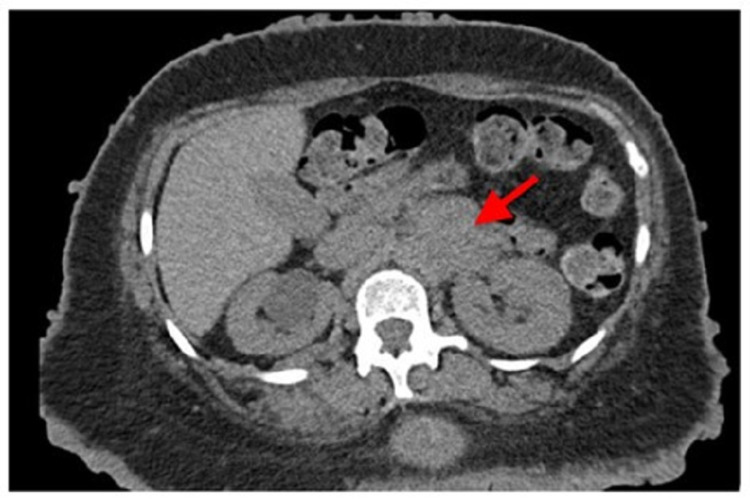
CT image demonstrating left adrenal mass (malignant peripheral nerve sheath tumor) measuring 5.5 cm × 3.9 cm.

**Figure 2:**
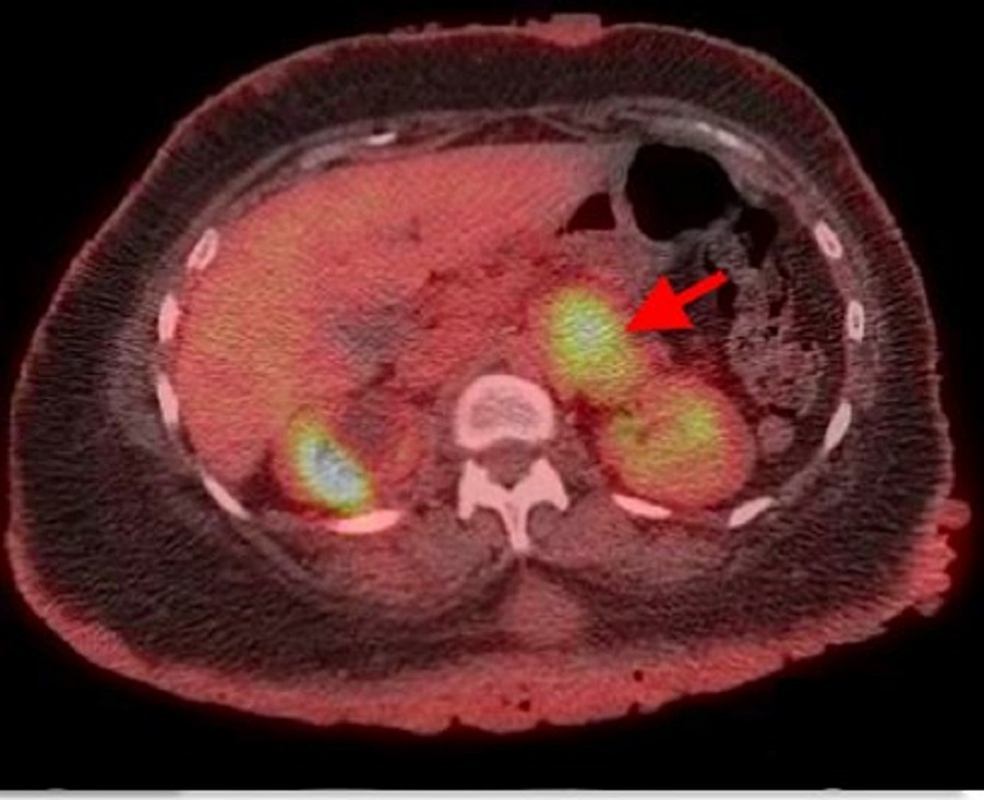
PET/CT demonstrating a mass (4.9 cm × 4.0 cm) in the medial aspect of the left kidney with hypermetabolic activity in the range of metastatic disease.

An extensive workup was performed by the medical team prior to the general surgery consultation. Five months earlier, she had presented to the emergency department with complaints of pain along her lateral chest wall, overlying the fourth and fifth ribs. Due to the patient’s history of breast cancer, a CT thorax was performed and demonstrated an incidental finding of a left adrenal mass measuring 5.5 cm × 3.9 cm (Figure 1) and a spiculated mass in the right lower lobe of the lung measuring 1.8 cm × 1.9 cm. Subsequently, PET/CT imaging was performed, which revealed a 1.7 cm × 1.6 cm nodule in the right lower lobe of the lung with borderline FDG activity (maximum SUV of 2.9) and a left adrenal mass with diffuse FDG activity (maximum SUV of 5.0; Figure 2). The oncology team decided to observe the lung nodule, given the borderline FDG activity. CT-guided biopsy of the abdominal mass was performed and showed neurofibroma with atypia.

A subsequent CT scan performed four months later demonstrated a left adrenal mass with heterogeneous contrast enhancement, which had increased in size (5.5 cm × 3.9 cm to 6.0 cm × 4.0 cm) since prior imaging. The patient developed worsening left-sided abdominal pain, which was uncontrollable with pain medications. At that time, the surgery team was consulted for evaluation and management of the mass.

Plasma and urine tests showed mildly elevated metanephrine and normetanephrine levels (85 pg/mL; reference range 0-62 and 187 pg/mL; reference range 0-145, respectively), low renin activity (0.278 ng/mL/hr; reference range 0.5-3.30), with normal serum aldosterone levels of <1.0 ng/dL (aldosterone renin ratio of 3.6 ng/dL). Potassium was within the normal range of 3.0-5.0 mMol/L.

Due to the mild elevation in urine metanephrines, pheochromocytoma was the main differential diagnosis of the adrenal mass. The patient was started on phenoxybenzamine preoperatively for two weeks before surgery. The patient was admitted prior to the planned surgery for the management of diet intolerance, fluid resuscitation, and repletion of electrolytes.

Open adrenalectomy was performed given the size and malignant features of the mass on imaging. Resecting the tumor was challenging and required careful dissection as the mass encased the left renal vessels and abutted the aorta. Blood pressure was successfully controlled throughout the four-hour operation despite constant tumor manipulation.

The mass measured 8 cm × 5.5 cm × 4.5 cm following resection. Pathology revealed a 114 g necrotic mass markedly adhered to the adrenal, but not involving it. The final pathology was consistent with a malignant peripheral nerve sheath tumor. Superior margins were found to be positive. The patient was observed following surgery and had an uneventful immediate postoperative recovery. She was discharged on postoperative day 7 with adequate pain control and improvement in her nausea and vomiting.

She presented two days following discharge with complaints of decreased oral intake, nausea, and diarrhea. CT imaging showed mild wall thickening of the small bowel and colon. Management focused on fluid resuscitation, repletion of electrolytes, and improving diet intolerance. Clostridioides difficile testing was negative, and an anti-diarrheal agent was started. Acute adrenal insufficiency was ruled out as the patient’s morning cortisol was within the reference range (19.6 µg/dL). The patient’s symptoms eventually resolved with conservative management.

One month following left adrenal mass resection, the patient remained asymptomatic with improvements in abdominal pain and nausea. Pathology was consistent with a malignant peripheral nerve sheath tumor with a positive superior margin. Due to the positive surgical margins on the final pathology, the patient was referred to medical oncology for discussion of adjuvant chemotherapy and radiation oncology for postoperative radiation. The patient was deemed a poor candidate for adjuvant radiation due to the high dose necessary and the proximity of normal tissue structures such as the small bowel. She was also deemed a poor candidate for systemic chemotherapy due to her functional status. On follow-up scans, approximately six months after surgery, the patient was found to have multiple foci of new metastatic disease, including liver, bone, and soft tissue. The patient was eventually transitioned to hospice.

## Discussion

MPNSTs are a rare, aggressive soft tissue tumor of peripheral nerve sheaths with an incidence of 0.1/100,000 per year [6]. The incidence of MPNSTs in patients with NF1 has been reported as 2% to 29% [6]. Approximately 50% of MPNSTs occur in patients with NF1, while the remainder of the cases is sporadic. MPNSTs are aggressive and tend to recur locally and metastasize hematogenously [6]. NF1-associated MPNSTs are associated with a poorer prognosis and lower five-year survival rates [5].

Clinical presentation varies widely based on the location of MPNSTs, but most will present with an enlarging mass and pain. Many patients will suffer from nerve-related symptoms such as paresthesia or weakness, which may be due to local compression or infiltration by the tumor [3,4].

Differentiating MPNST from neurofibromas by means of imaging studies is challenging, as MPNSTs commonly present as non-specific soft tissue masses [3]. On computed tomography, MPNSTs will have low attenuation due to their high lipid content [3]. MRI is the preferred study as it can help distinguish the tumor from its surrounding anatomy, which is helpful for surgical planning [4]. Wasa et al. [7] recently defined four magnetic resonance features to aid in distinguishing MPNST from neurofibromas: an increase in the largest dimension of the mass; the presence of a peripheral enhanced pattern; the presence of perilesional edema-like zone; and the presence of intratumoral cystic lesions. Identifying two or more of these characteristics is suggestive of MPNST (specificity of 90%, sensitivity of 61%) and suggests biopsy should be done to further aid in diagnosis. On PET imaging, MPNSTs are FDG-avid with an average SUV of 8.5 [3].

MPNSTs are aggressive tumors and pose a significant challenge with regard to available and effective treatment strategies. The mainstay of treatment is surgical resection with negative margins; however, MPNSTs tend to recur locally. In one case series by Anghileri et al. [6], local recurrence and/or distant metastasis were seen in 30% of patients at 10 years post-surgical resection. Patients with a positive surgical margin had a 2.4-fold risk of developing a local recurrence [6]. Chemotherapy and radiotherapy as adjunctive treatments to surgery continue to be investigated. Several chemotherapeutic agents are currently being trialed for MPNSTs, including erlotinib, sorafenib, imatinib, bevacizumab/everolimus, and Gantespib/Sirolimus [5]. Radiotherapy is currently recommended for all patients with large MPNSTs and positive surgical margins, although there are no current studies that show improvement in overall survival with the addition of radiation therapy [5].

Although the association between NF1 and MPNST has been well established, recent research suggests that the lifetime risk of MPNST in NF1 is higher than previously anticipated, warranting more vigilant surveillance and a low threshold for workup [8]. Furthermore, studies have shown that MPNST is often misdiagnosed [9]. MPNSTs arise from pre-existing plexiform tumors. Recent studies have revealed that most NF1 patients develop MPNSTs in deeper locations, with only a small quantity occurring in classic cutaneous plexiform tumors [8,10].

## Conclusions

This case highlights an important differential diagnosis of tumors in patients with NF1. Our patient’s history of NF1 and tests revealing increased urine catecholamines lead us to suspect pheochromocytoma as the principal diagnosis. This guided our treatment in prescribing the patient alpha blockers prior to surgical resection. Although our choice for surgical resection aligned with guidelines for the management of MPNSTs, it is possible that a more meticulous analysis of imaging may have led to earlier recognition of the MPNST. Finally, our case exhibits the necessity of a multidisciplinary team of surgeons, internists, oncologists, radiologists, and pathologists in diagnosing and treating NF1 patients with an MPNST to ensure safe and effective treatment.
